# A novel “six stitches” procedures for pediatric and adult buried penis

**DOI:** 10.1590/S1677-5538.IBJU.2017.0688

**Published:** 2019

**Authors:** Junhao Lei, Chunhua Luo, Xinghuan Wang, Xinjun Su

**Affiliations:** 1 Department of Urology, Zhongnan Hospital of Wuhan University, Wuhan University, Wuhan, China;; 2 Center for Evidence-based and Translational Medicine, Wuhan University, Wuhan, China

## Abstract

**Introduction:**

The buried penis, if not treated before adolescence, will lead to psychological and physical disorders in adulthood. Therefore, early surgical intervention is necessary. At present, the common surgical methods include the penile corpus fixation, the Johnson's operation, the Devine's method, the modified Devine's method, Shiraki's method, etc. However, we found that these traditional surgeries showed various postoperative complications, such as long-term prepuce edema, avascular necrosis of skin flaps, stenotic prepuce, scarring, and poor appearance. This video shows the main technical steps of our innovative surgical procedure “Six Stitch” (SS) method for the buried penis.

**Materials and Methods:**

The designation of the so-called SS method was based on the total knots made (six knots were made for the SS procedure).

After the crura penis was fully exposed via a longitudinal incision at the penoscrotal junction, at the 2 o'clock position (around the penis), the superficial layer of albuginea of the crura penis was sutured to the prepubic ligament with 2-0 non-absorbable sutures to prevent the retraction of the penis (the 1^st^ knot). The same procedure was used for the 10 o'clock position (the 2^nd^ knot); At the 2 o'clock position, the skin and subcutaneous tissue at the pubic mound were sutured to the prepubic ligament to reconstruct the appearance of dorsum penis (the 3^rd^ knot). The same procedures were used for the 10 o'clock position (the 4^th^ knot). At the 5 o'clock position, the ventral albuginea was sutured to the tunica dartos and subcutaneous tissue at the penoscrotal junction to reconstruct the penoscrotal angle (the 5^th^ knot). The same procedures were used for the 4 o'clock position (the 6^th^ knot). Finally, the gloved prepuce was reset and circumcision was conducted if the redundant prepuce existed.

**Results:**

We have done a total of 64 cases of SS procedures for concealed penis; mean length improvement was 3.8 ± 0.5 cm, with a satisfying 95 percent (61 / 64), which was much longer than the outcome of the above-mentioned methods. Mean operative time was 62.3 ± 12.1 minutes, and there was no serious intraoperative or postoperative complication (only 2 presented scar hyperplasia at the incision site).

**Conclusions:**

In conclusion, after the SS procedure, patients with buried penis can acquire an almost 4 cm improvement of penile length and covert incision at the midline of the scrotum, with an acceptable and low incidence of adverse events. This safe and effective procedure may be a viable option for the surgical management of pediatric and adult buried penis.

## ARTICLE INFO

**Available at: http://www.intbrazjurol.com.br/video-section/20170688_ Lei_et_al**

**Int Braz J Urol. 2019; 45 (video #2): 190-1**

